# Natural products modulate renal metabolic reprogramming in diabetic kidney disease

**DOI:** 10.1515/jtim-2026-0050

**Published:** 2026-08-12

**Authors:** Wenru Wang, Han Zhu, Keqin Zhao, Xinhui Wang, Ying Liang, Jianlong Xu, Xiaobei Ma, Renhuan Yu, Peng Liu

**Affiliations:** Department of Nephrology, Xiyuan Hospital of China Academy of Chinese Medical Sciences, Beijing, China; Institute of Basic Theory for Chinese Medicine, China Academy of Chinese Medical Sciences, Beijing, China

**Keywords:** diabetic kidney disease, metabolic reprogramming, natural products, mitochondrial dysfunction, traditional Chinese medicine

## Abstract

Diabetic kidney disease (DKD) is a major cause of end-stage renal disease worldwide. Its pathogenesis is highly complex and involves dysfunction across multiple renal cell types and metabolic pathways, thereby underscoring the urgent need for novel therapeutic strategies. In recent years, renal metabolic reprogramming has been increasingly recognized as a central driver of DKD progression. This review systematically examines the mechanisms of metabolic reprogramming in different renal cell types and highlights their contribution to renal injury. Particular emphasis is placed on the ability of natural products (including glycosides, terpenoids, flavonoids, alkaloids, and polysaccharides) to confer renal protection by modulating key regulatory nodes of metabolic reprogramming. Through these mechanisms, natural products enhance mitochondrial function, suppress aberrant glycolysis, and improve hypoxia-related responses. Collectively, these findings not only underscore the therapeutic potential of natural products in targeting renal metabolic reprogramming but also provide a theoretical foundation and potential molecular targets for the development of novel DKD treatment strategies.

## Introduction

Diabetic kidney disease (DKD), a major complication of diabetes, is a leading cause of end-stage renal disease globally. Affecting 30%–40% of patients with diabetes, DKD imposes a substantial socioeconomic burden, with associated healthcare expenditures having increased by over 300% in the last two decades.^[1, 2, 3]^ The pathogenesis is complex, mainly initiated by systemic insults such as hyperglycemia-induced glucotoxicity and persistent low-grade inflammation.^[4, 5]^ It is also related to genetic and other factors.^[6, 7]^ These initial triggers precipitate a cascade of intrarenal damage, including mitochondrial dysfunction,^[3]^ podocyte injury,^[8]^ and autophagic dysregulation,^[9]^ which is further exacerbated by genetic susceptibility,^[10]^ epigenetic modifications,^[11]^ and gut dysbiosis.^[12]^ These convergent pathways culminate in the progressive deterioration of renal structure and function. Central to this process is the concept of metabolic reprogramming, now recognized as a fundamental driver of DKD progression.^[13]^

Metabolic reprogramming is a dynamic process wherein cells recalibrate their energy metabolism to meet the demands of survival and proliferation.^[14]^ Hallmarks of this phenomenon include enhanced glycolysis, altered expression of key metabolic enzymes, and modified substrate utilization for amino and fatty acid metabolism.^[15]^ Healthy cells primarily rely on mitochondrial oxidative phosphorylation (OXPHOS) for efficient, high-yield ATP production from diverse substrates.^[16]^ In contrast, glycolysis offers a rapid but lower-yield ATP supply, a trade-off that is crucial for acute stress responses and specialized cellular functions.^[17]^ In diseases such as DKD, however, cells undergo a pathological shift from OXPHOS toward an adaptive, yet ultimately detrimental, reliance on glycolysis.^[18]^ This metabolic rewiring is not merely an adaptation but a direct contributor to DKD progression, as it exacerbates oxidative stress, inflammation, and mitochondrial dysfunction.^[19]^

In the hyperglycemic milieu of DKD, metabolic reprogramming drives lipotoxicity, glucotoxicity, and mitochondrial dysfunction, thereby accelerating pro-inflammatory and fibrotic cascades. In renal tubular epithelial cells, for example, a pathological switch from fatty acid oxidation (FAO) to glycolysis is compounded by increased CD36-mediated lipid uptake and CPT1 inhibition. The resulting lipid accumulation triggers lipotoxicity, oxidative stress, and apoptosis.^[20, 21]^ Concurrently, this glycolytic shift generates lactate and an acidic microenvironment. This environment, in turn, activates deacetylases such as Sirt5, promoting the secretion of pro-inflammatory and pro-fibrotic factors that drive tubulointerstitial fibrosis.^[22, 23]^ A similar metabolic dysregulation unfolds within the glomerulus. Thus, cell-specific metabolic reprogramming converges to establish a self-reinforcing cycle of glucotoxicity, lipotoxicity, and inflammation that propels the progression of DKD. DKD patients also exhibit different metabolic phenotypes and different clinical trajectories. Recent metabolomic studies have identified distinct metabolic subgroups among DKD patients, revealing significant inter-individual differences in pathways such as FAO, amino acid metabolism, and energy utilization.^[24]^ Given this heterogeneity, therapeutic strategies aimed at metabolic reprogramming may yield different outcomes across patient subgroups.

Standard-of-care pharmacotherapies, including angiotensin-converting enzyme (ACE) inhibitors and sodium-glucose cotransporter 2 (SGLT2) inhibitors, can slow DKD progression, yet their efficacy is limited by adverse effects such as hyperkalemia and acute kidney injury, and they fail to halt the disease trajectory completely. These limitations, compounded by the challenges of long-term polypharmacy, underscore the urgent need for new therapeutic paradigms. Natural products, many derived from traditional Chinese medicine (TCM), offer a compelling alternative, characterized by multi-target mechanisms and favorable safety profiles.^[25, 26, 27, 28]^ For instance, salidroside has been found to reverse tubulointerstitial fibrosis by targeting the TGF-*β*1/Smad signaling axis and to mitigate hypoxia-induced podocyte pyroptosis.^[29]^ Similarly, anthocyanins delay glomerular lesion progression in db/db mice by modulating amino acid metabolism and inhibiting extracellular matrix (ECM) deposition.^[30]^ Collectively, these examples demonstrate that the pleiotropic actions of natural products—encompassing antioxidant, anti-fibrotic, and metabolic-reprogramming effects—establish a strong rationale for their integration into novel therapeutic strategies for DKD.^[31, 32, 33]^ The combination of natural products with existing standard treatments also shows great potential. Meta-analyses have shown that the combination of Jinshuibao and ACEI/ARB is more effective than monotherapy,^[34]^ and animal experiments have also shown that puerarin combined with cargagliflozin significantly reduces renal lipid accumulation.^[35]^ However, the combination of natural products also poses potential safety risks such as drug interactions and metabolic interference, and the potential nephrotoxicity and adverse reaction risks of some herbs need to be carefully evaluated.^[36]^ At the same time, the effectiveness of this efficacy is also influenced by the heterogeneity of metabolic phenotypes in patients. The recently reported natural product stachyose further supports the feasibility of precisely matching drugs by metabolic phenotype by stabilizing SGLT2 and reversing metabolic reprogramming and fibrosis of the proximal tubule.^[37]^

**Figure 1:**
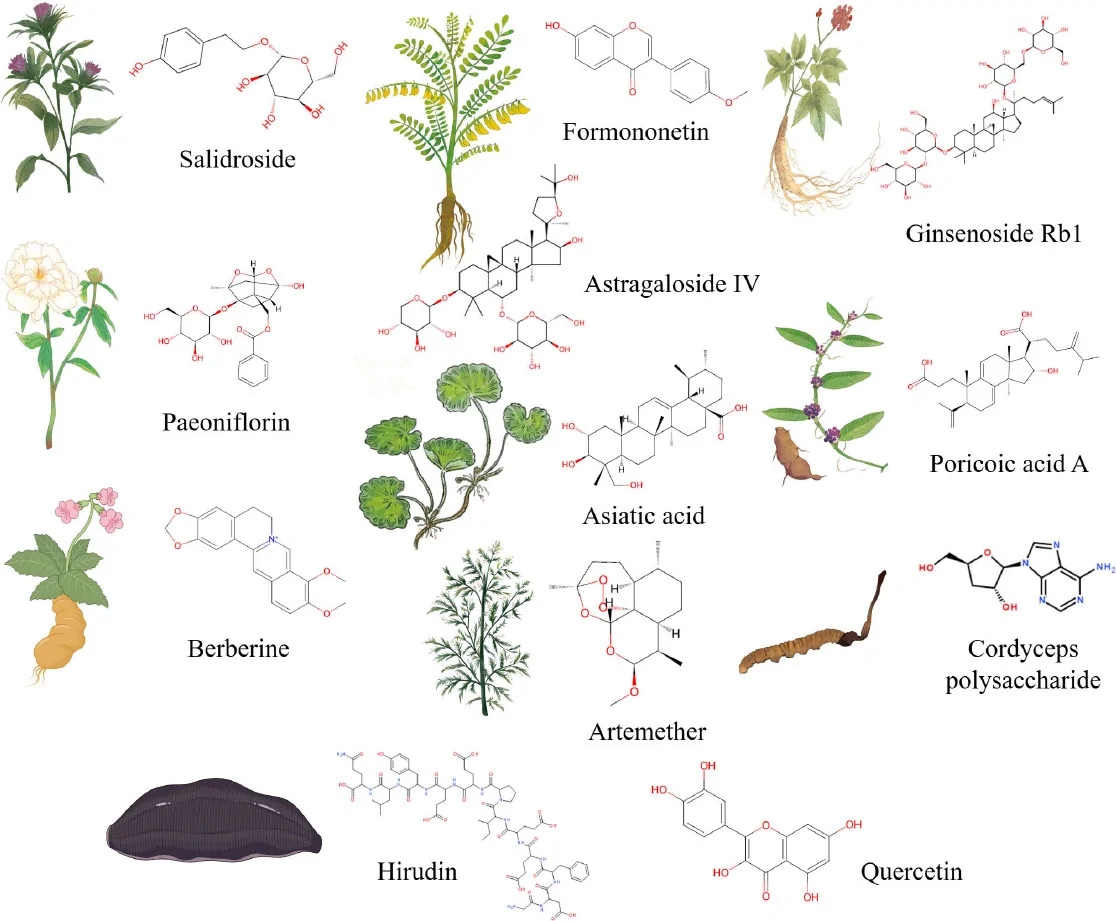
Illustration of various natural products and their corresponding chemical structures that exhibit therapeutic potential in the treatment of diabetic kidney disease. Each natural product is paired with a visual representation of the plant from which it is derived, alongside its chemical structure. The image is from the Figdraw.com, and the composite structure is from the PubChem database.

Here, we synthesize the evidence that natural products (Figure 1) can correct pathological metabolic reprogramming in DKD. We argue that by modulating these core metabolic pathways, such multi-target compounds represent a novel therapeutic strategy to overcome the limitations of current pharmacotherapies.

## Mechanisms of metabolic reprogramming in different renal cells in diabetic kidney disease

Renal cell populations exhibit distinct metabolic phenotypes. Glomerular cells—including endothelial cells, mesangial cells, and podocytes—rely primarily on glycolysis and OXPHOS,^[38, 39]^ whereas the high-energy demands of proximal tubular cells (PTCs) are *met almost exclusively* by FAO.^[40]^ The primary function of the glomerulus is to act as a physical barrier rather than to engage in large-scale active transport of substances.^[41]^ Its energy consumption is mainly used for maintaining the cytoskeleton (especially the structure of podocyte foot processes), protein synthesis, and cell signaling transduction, which are “housekeeping” functions. These tasks require relatively low energy but have a higher demand for the immediacy of ATP supply.^[38, 42]^ Glycolysis and oxidative phosphorylation are well-suited to meet the energy needs of glomerular cells. In diseases primarily characterized by glomerular injury, such as diabetic nephropathy, the key is to inhibit the abnormal glycolysis driven by hyperglycemia and its downstream pathological pathways.^[43]^ Proximal tubule cells undertake the most demanding task of reabsorption in the kidney, where approximately 65%–70% of the filtrate (water, sodium, chloride, glucose, amino acids, *etc*.) is reabsorbed back into the bloodstream.^[44]^ This task requires a substantial and continuous supply of energy, and FAO is the optimal choice to meet this demand.^[45, 46]^ Restoring or enhancing the impaired FAO capacity of PCT cells is an attractive therapeutic direction in both acute kidney injury and CKD.^[47, 48]^ In DKD, this metabolic specialization collapses.^[13]^ Mitochondrial dysfunction emerges not merely as an energy deficit but as a central signaling hub that orchestrates a pathological reprogramming of cellular metabolism, which drives a unifying shift from efficient OXPHOS toward aerobic glycolysis—a metabolic phenotype analogous to the Warburg effect observed in cancer.^[13]^ While this glycolytic switch may serve as an acute survival mechanism, its persistence in the chronic milieu of DKD becomes maladaptive, fueling progressive renal injury.^[49, 50]^

Mitochondrial dysfunction is not merely a consequence but a primary driver of DKD, initiating the cascade of metabolic reprogramming.^[51]^ Mitochondrial dysfunction is closely related to mitochondrial quality control (MQC), and adjusting MQC can effectively improve cell damage and apoptosis caused by imbalances in energy metabolism.^[52]^ Consequently, the severity of renal injury reflects the balance between persistent mitochondrial damage inflicted by the hyperglycemic state and the efficacy of compensatory MQC mechanisms.^[53, 54]^

MQC is essential for cellular homeostasis, relying principally on mitophagy—the selective autophagic clearance of damaged mitochondria—to maintain a healthy organellar pool.^[55, 56]^ When this critical clearance mechanism is impaired, it can lead to severe pathological consequences.^[57]^ For example, in DKD, studies have found that it is the impairment of mitophagy that leads to the accumulation of dysfunctional mitochondria in the kidneys, ultimately exacerbating the progression of the disease. Targeting this pathological mechanism, studies have confirmed the effects of specific pharmacological interventions.^[58]^ For example, the SGLT2 inhibitor empagliflozin can restore autophagy/mitophagy in the heart and inhibit mitochondrial fission, thereby improving MQC and exerting a protective effect in a model of diabetic myocardial infarction. This process is orchestrated by the PTEN-induced kinase 1 (PINK1)/Parkin pathway, which tags dysfunctional mitochondria for degradation following their segregation from the network *via* fission.^[59]^

However, in the diabetic state, this MQC mechanism fails, primarily due to mitochondrial dysfunction caused by the sustained metabolic burden on the kidneys in diabetes, leading to oxidative stress and functional disturbances.^[60]^ A key feature of this dysfunction is the imbalance of mitochondrial dynamics, characterized by a shift of the mitochondrial network toward excessive fission and fragmentation.^[61]^ Experimental evidence shows that this mitochondrial fragmentation occurs before the clinical signs of kidney damage appear, confirming its role as a primary initiator of the disease.^[62]^ At the molecular level, hyperglycemia can phosphorylate and activate the fission protein Drp1 by activating the ROCK1 signaling pathway, directly triggering excessive mitochondrial fission.^[62]^ Meanwhile, in tubular cells, upregulated myo-inositol oxygenase not only exacerbates mitochondrial fission but also inhibits the autophagy process used to clear damaged mitochondria, further disrupting the MQC system.^[63]^ In contrast to these damaging mechanisms, a protective pathway has also been identified: the Rap1b protein, which is downregulated in DKD, can promote mitochondrial health and inhibit excessive fission by activating the PGC-1*α* signaling cascade, thereby effectively reducing tubular injury.^[64]^ Regarding mitophagy, downregulation of the key protein TXNIP in renal tubules can inhibit the mTOR signaling pathway, thereby correcting mitophagy.^[65, 66]^ By correcting the dual effects of NR4A1, which promotes excessive mitochondrial fission and inhibits mitochondrial clearance, and by activating FoxO1 to correct the PINK/Parkin pathway and enhance mitophagy, podocyte damage can be alleviated and disease progression can be mitigated.^[67, 68]^ Therefore, restoring mitophagic flux is a key therapeutic strategy to halt disease progression. Next, we will explore the mechanisms triggering metabolic reprogramming in various renal cells, focusing on mitochondrial dysfunction, enhanced glycolysis, and hypoxia.

### Tubular epithelial cells

**Figure 2:**
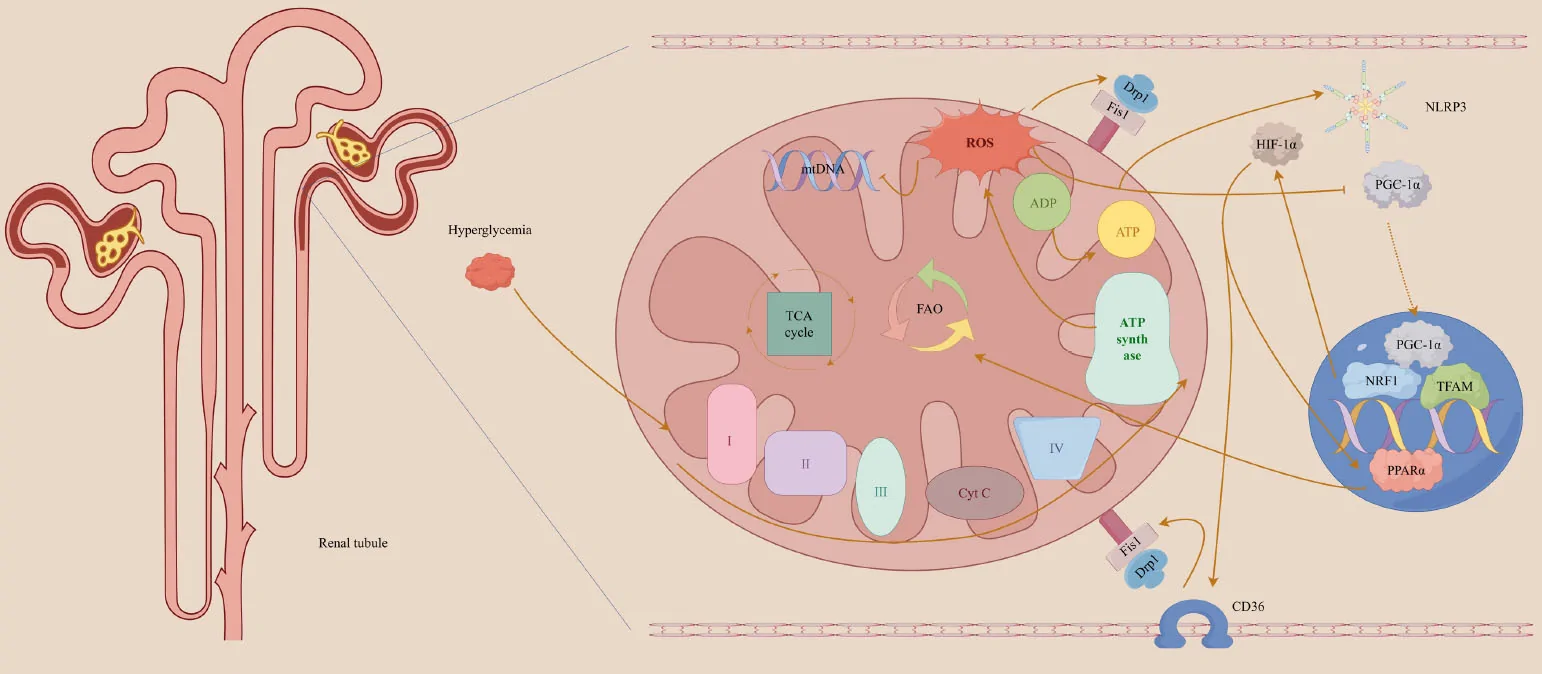
Chronic hyperglycemia drives a switch in RTECs from fatty-acid oxidation (FAO)-coupled oxidative phosphorylation to a dysfunctional mitochondrial state. Excess glucose increases mitochondrial reactive oxygen species (ROS), damages mitochondrial DNA (mtDNA), and disrupts the electron transport chain (Complexes I–IV and cytochrome c), resulting in inefficient ATP synthase activity, reduced ATP generation, and an altered ADP/ATP ratio. FAO and tricarboxylic acid (TCA) cycle flux are perturbed, while CD36-mediated lipid uptake augments lipotoxic stress. Mitochondrial dynamics favor fission through dysregulated Drp1/Fis1, and mitonuclear signaling is impaired by downregulation of the PPAR*α*-PGC-1*α*-NRF1/TFAM axis, which suppresses mitochondrial biogenesis and *β*-oxidation programs. In parallel, activation of HIF-1*α* and the NLRP3 inflammasome promotes inflammatory and fibrotic responses. Together, these changes culminate in energy deficiency, tubular injury, and progression of diabetic nephropathy. RTECs: renal tubular epithelial cells; FAO: fatty acid oxidation; ROS: reactive oxygen species; mtDNA: mitochondrial DNA; TCA: tricarboxylic acid; ATP/ADP: adenosine tri/di-phosphate; Cyt C: cytochrome c; Drp1: dynamin-related protein 1; Fis1: mitochondrial fission 1 protein; HIF-1*α*: hypoxia-inducible factor-1 alpha; NLRP3: NOD-like receptor protein 3; PGC-1*α*: peroxisome proliferator-activated receptor-*γ* coactivator-1*α*; NRF1: nuclear respiratory factor 1; TFAM: mitochondrial transcription factor A; PPAR*α*: peroxisome proliferator-activated receptor-*α*; CD36: cluster of differentiation 36.

PTCs have immense energy demands, as they are responsible for reabsorbing nearly all solutes from the glomerular filtrate. This metabolic workload is fueled almost exclusively by mitochondrial FAO, not glycolysis.^[49]^ This profound reliance on FAO, however, renders PTC mitochondria exceptionally vulnerable to the glucotoxic, lipotoxic, and pro-inflammatory environment of DKD, making them the primary and earliest sites of cellular damage (Figure 2).^[69]^

#### Mitochondrial dysfunction

In DKD, the immense task of reabsorbing excess glucose triggers a vicious cycle of mitochondrial collapse in PTCs.^[70]^ This workload forces mitochondria into overdrive, overwhelming the electron transport chain (ETC) and culminating in a surge of ROS.^[71]^ This ROS surge then launches a multi-pronged assault on the mitochondrial network.

First, ROS directly damages mtDNA, which impairs the synthesis of ETC complexes I and III and cripples electron transport efficiency.^[72]^ Second, ROS—alongside glucotoxicity and inflammatory cytokines—suppresses PGC-1*α*, the master regulator of mitochondrial biogenesis.^[73]^ This suppression inactivates downstream targets, including nuclear respiratory factors and mitochondrial transcription factor A (TFAM), effectively halting the production of new, functional mitochondria.^[56, 74]^ Third, in the pathogenesis of DKD, mitochondrial damage mediated by dynamin-related protein 1 (Drp1) represents a critical step in podocyte dysfunction, with excessive ROS production acting as a major pathogenic factor in this process.^[75]^ Under conditions of elevated free fatty acids, Drp1 activation triggers excessive mitochondrial fission and dysfunction. This dysfunction, in turn, directly promotes ROS overproduction, ultimately leading to podocyte apoptosis. Furthermore, receptor-interacting protein kinase 3 (RIPK3) has been identified as an important upstream regulator of Drp1 activation. Through the PGAM5-Drp1 signaling axis, RIPK3 initiates a pathological cascade characterized by mitochondrial fission, dysfunction, and excessive ROS generation.^[76]^

The therapeutic potential of reversing this pathology is underscored by studies using P110, a selective Drp1 inhibitor. By blocking excessive Drp1-mediated fission, P110 restores AMPK/PGC-1*α*/TFAM signaling, enhances mitochondrial biogenesis, and rescues metabolic function in tubular cells from DKD patients.^[77]^

#### Glycolysis

The mitochondrial energy crisis in DKD triggers a maladaptive metabolic switch in PTCs, forcing them to abandon their physiological reliance on FAO in favor of excessive glycolysis.^[78]^ This reprogramming is orchestrated largely by hypoxia-inducible factor-1 *α* (HIF-1*α*), a transcription factor that is overexpressed in DKD and actively suppresses the FAO machinery by dysregulating key targets like peroxisome proliferator-activated receptor *α* (PPAR*α*).^[79, 80, 81]^ This glycolytic shift is further amplified by the upregulation of the fatty acid translocase CD36, which redirects cellular metabolism away from FAO.^[82, 83]^

This enforced glycolysis initiates a cascade of cellular pathologies. It leads to the accumulation of intracellular lipids and lactate, which itself drives mitochondrial fragmentation *via* lactylation of the fission protein Fis1, thus exacerbating ROS production and mitochondrial dysfunction.^[84, 85]^ Simultaneously, the metabolic flux from heightened glycolysis provides the substrate for the secretion of pro-inflammatory and pro-fibrotic factors, while also fueling the activation of the NLRP3 inflammasome, collectively driving tubulointerstitial inflammation, apoptosis, and fibrosis.^[86]^

Ultimately, the combination of mitochondrial failure and chronic glycolytic stress drives PTCs into a state of premature senescence, characterized by the acquisition of a senescence-associated secretory phenotype.^[87, 88]^ This pro-fibrotic secretome establishes a self-perpetuating cycle of “maladaptive repair”, relentlessly accelerating the progression toward end-stage renal disease.

### Podocytes

**Figure 3:**
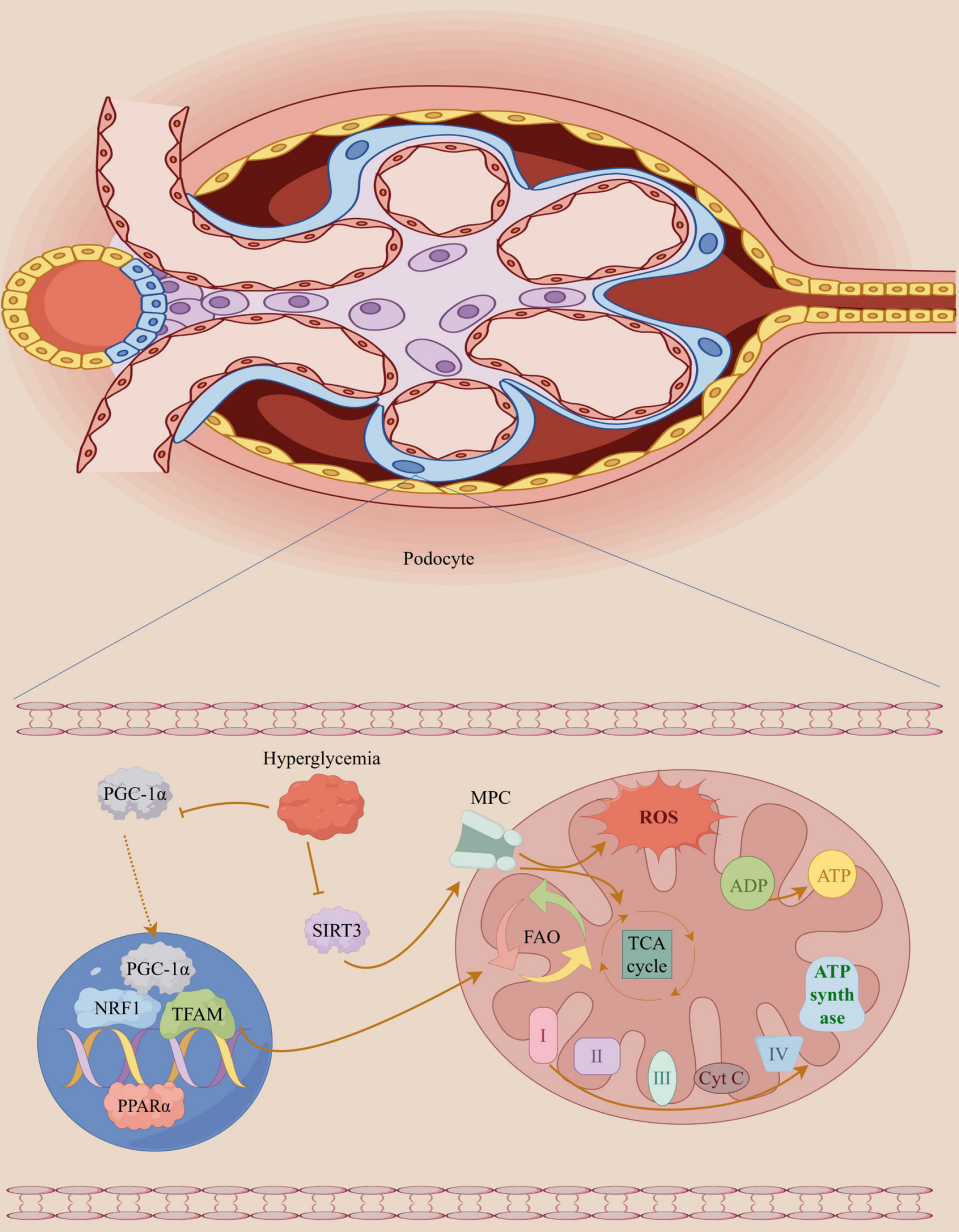
Persistent hyperglycemia remodels podocyte metabolism toward mitochondrial dysfunction. In the mitochondrion, tricarboxylic acid (TCA) cycle flux and fatty-acid oxidation (FAO) are perturbed, electron-transport chain Complexes I–IV and cytochrome c (Cyt C) are inefficient, and ATP synthase activity declines, resulting in reduced ATP output and an altered ADP/ATP ratio. Excessive reactive oxygen species (ROS) further damages mitochondrial components. Hyperglycemia represses SIRT3 and attenuates mitochondrial pyruvate carrier (MPC)-dependent substrate delivery, thereby limiting oxidative metabolism. In parallel, downregulation of the PGC-1*α*-NRF1/TFAM transcriptional axis reduces mitochondrial biogenesis and *β*-oxidation programs, in part via impaired PPAR*α* signaling. These metabolic defects compromise podocyte homeostasis and contribute to foot process injury and the progression of diabetic kidney disease. ROS: reactive oxygen species; FAO: fatty-acid oxidation; TCA: tricarboxylic acid; Cyt C: cytochrome c; ATP/ADP: adenosine tri/di-phosphate; MPC: mitochondrial pyruvate carrier; SIRT3: sirtuin-3; PGC-1*α*: peroxisome proliferator-activated receptor-*γ* coactivator-1*α*; NRF1: nuclear respiratory factor 1; TFAM: mitochondrial transcription factor A; PPAR*α*: peroxisome proliferator-activated receptor-*α*.

In the early stages of diabetic nephropathy, the hyperglycemic environment directly induces the production of ROS in podocytes and leads to their apoptosis, which is the fundamental cause of disease initiation (Figure 3). As the disease progresses, the local hypoxic environment in the kidneys activates HIF-1. This factor first inhibits the expression of PPAR*α*, a key regulator of FAO,^[89, 90]^ and then directly suppresses the activity of key enzymes in mitochondria, such as MCAD and LCAD,^[91]^ thereby forcibly shifting the energy metabolism mode of podocytes and surrounding renal tissues from efficient FAO to glycolysis. This HIF-1*α*-mediated metabolic reprogramming has been proven to be the core link leading to lipid accumulation and functional deterioration in the kidneys of diabetic patients, and SGLT2 inhibitors can reverse this process by inhibiting HIF-1*α*, thereby exerting protective effects on renal units, including podocytes.^[92]^ Eventually, in the late stages of the disease, glomerulosclerosis caused by metabolic disorders and long-term damage exerts continuous mechanical stress on podocytes, further activating the yes-associated protein (YAP) signaling pathway within them. This pathway promotes the expression of fibrosis-related proteins, exacerbating the structural damage to the kidneys, creating a vicious cycle, and ultimately driving the disease towards end-stage.^[93]^

This cellular collapse is rooted in mitochondrial failure, where a hyperglycemic environment triggers a cascade of metabolic derangements. A key initiating event is the downregulation of the mitochondrial deacetylase Sirt3, which leads to the hyperacetylation of mitochondrial pyruvate carrier 2 (MPC2).^[94]^ This modification cripples pyruvate import into the mitochondrial matrix, precipitating a bioenergetic crisis characterized by ROS accumulation, diminished ATP production, and the loss of mitochondrial membrane potential (MMP), ultimately driving podocyte apoptosis.^[95, 96, 97]^ Concurrently, hyperglycemia suppresses PGC-1*α*, impairing the cell's capacity for FAO. The convergence of these metabolic insults—impaired pyruvate metabolism and defective FAO—creates an unsustainable cellular environment that accelerates podocyte injury and glomerulosclerosis.

### Glomerular cells

Glomerular cells—including podocytes, endothelial cells, and mesangial cells—exhibit a distinct metabolic signature. Compared to the oxidative powerhouse of the proximal tubules, these cells have a lower oxidative capacity and are fueled primarily by glucose oxidation to meet their basal energy demands.^[96]^ In DKD, however, this glucose-dependent metabolism becomes a critical vulnerability.

The hyperglycemic environment triggers pathological reprogramming at multiple, interacting levels. At the metabolic level, it drives overactivation of the pentose phosphate pathway, which paradoxically generates an excess of NADPH that exacerbates ROS production, fueling glomerular fibrosis and inflammation.^[97]^ This is coupled with the aberrant reactivation of signaling cascades: in mesangial cells, endothelin-1 triggers pro-hypertrophic ERK and PI3K signaling,^[98]^ while in podocytes, reactivation of the Notch-1 pathway promotes podocytopathy.^[99]^

This reprogramming also extends to the epigenetic level. In podocytes, hyperglycemia drives the overexpression of histone deacetylase 4 (HDAC4), which strips the critical slit diaphragm protein nephrin of its protective acetylation, targeting it for degradation.^[100]^ This mechanism highlights a potential therapeutic axis, as interventions that upregulate miR-29a can stabilize nephrin by suppressing HDAC4.^[101]^ The fundamental link between metabolic flux and structural integrity is further underscored by findings that altering renal glucose transport, even in a non-diabetic state, induces glomerular contraction, revealing a new perspective on the structural consequences of metabolic dysregulation in DKD.^[102]^

**Figure 4:**
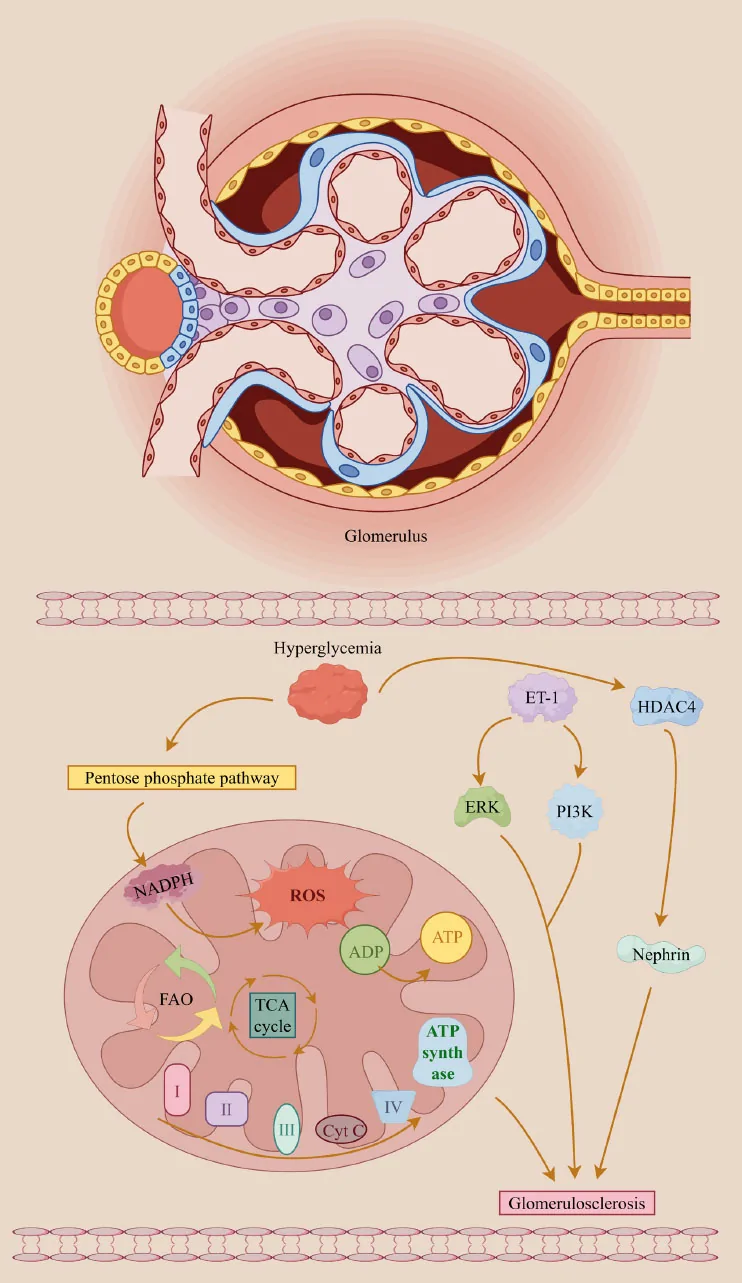
Chronic hyperglycemia induces coordinated metabolic and signaling alterations across glomerular cells (podocytes, endothelial and mesangial cells). Mitochondria exhibit disturbed fatty-acid oxidation (FAO) and tricarboxylic acid (TCA) cycle flux with inefficient electron-transport chain activity (Complexes I–IV and cytochrome c), resulting in reduced ATP synthase output and an altered ADP/ATP ratio. Excess reactive oxygen species (ROS) accumulate, in part fueled by increased pentose phosphate pathway (PPP)–derived NADPH that supports NADPH-oxidase activity. In parallel, hyperglycemia elevates endothelin-1 (ET-1), which activates PI3K and ERK cascades, and promotes epigenetic repression via HDAC4. These pathways converge on the slit diaphragm protein nephrin, compromising filtration barrier integrity. The combined metabolic and transcriptional reprogramming accelerates extracellular matrix deposition and capillary injury, culminating in glomerulosclerosis. PPP: pentose phosphate pathway; NADPH: nicotinamide adenine dinucleotide phosphate (reduced); ROS: reactive oxygen species; FAO: fatty-acid oxidation; TCA: tricarboxylic acid; Cyt C: cytochrome c; ATP/ADP: adenosine tri/di-phosphate; ET-1: endothelin-1; PI3K: phosphoinositide 3-kinase; ERK: extracellular signal-regulated kinase; HDAC4: histone deacetylase 4.

Thus, glomerulosclerosis in DKD is not a single pathological event but the cumulative result of multi-layered reprogramming—spanning metabolic flux, signaling networks, and epigenetic control—that corrupts the function of the entire glomerular unit (Figure 4).^[103]^

### Macrophages

**Figure 5:**
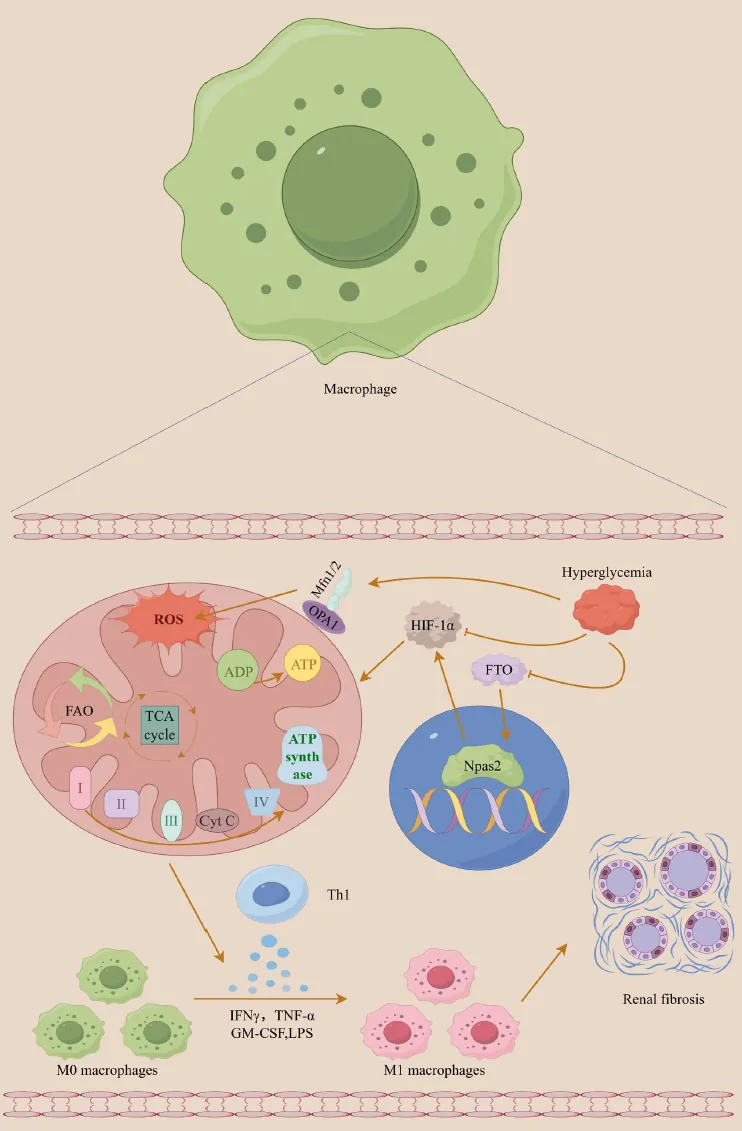
Under hyperglycemic conditions, macrophages undergo mitochondrial metabolic remodeling characterized by enhanced reactive oxygen species (ROS) generation, disturbed tricarboxylic acid (TCA) cycle flux, reduced fatty-acid oxidation (FAO), and inefficient electron-transport–coupled ATP synthesis (Complexes I–IV, cytochrome c, ATP synthase), which together shift cellular metabolism toward a pro-inflammatory state. Mitochondrial dynamics are altered with imbalance of the fusion regulators OPA1 and Mfn2. Hyperglycemia also stabilizes HIF-1α and, together with the m6A demethylase FTO, transcriptionally upregulates the circadian factor Npas2, reinforcing inflammatory gene programs. Th1-derived cues—including IFN-γ, TNF-α, and GM-CSF, as well as bacterial LPS—drive polarization of resting M0 macrophages toward M1 macrophages. The combined metabolic and transcriptional reprogramming amplifies cytokine production and promotes renal interstitial matrix deposition, culminating in renal fibrosis. ROS: reactive oxygen species; TCA: tricarboxylic acid; FAO: fatty-acid oxidation; Cyt C: cytochrome c; ATP/ADP: adenosine tri/di-phosphate; OPA1: optic atrophy protein 1; Mfn2: mitofusin-2; HIF-1α: hypoxia-inducible factor-1 alpha; FTO: fat mass and obesity-associated protein; Npas2: neuronal PAS domain protein 2; Th1: T helper 1 cell; IFN-γ: interferon-gamma; TNF-α: tumor necrosis factor-alpha; GM-CSF: granulocyte-macrophage colony-stimulating factor; LPS: lipopolysaccharide; M0/M1: resting/classically activated macrophage.

In the pathogenesis of diabetic nephropathy, the hyperglycemic environment first directly induces injury to glomerular mesangial cells and podocytes, which is the initiating factor for subsequent pathological reactions.^[104]^ This initial cellular damage activates inflammatory pathways in the kidneys, leading to massive macrophage infiltration, which is considered a key pathological feature driving the progression of diabetic nephropathy (Figure 5).^[105]^ However, the infiltrating macrophages do not simply exacerbate damage; there are complex regulatory mechanisms within them. Studies have found that the cyclooxygenase-2 (COX-2) pathway expressed by macrophages themselves can promote their polarization towards the M2 phenotype, which has tissue repair functions, thereby exerting a protective effect on the kidneys. This reveals a dynamic balance between pro-inflammatory and anti-inflammatory roles of macrophages.^[106]^ The importance of this inflammatory pathway is also indirectly confirmed: in models simulating the action of SGLT2 inhibitors, even with glucosuria, the kidneys do not show damage because they avoid infiltration of inflammatory cells, including macrophages, suggesting that blocking the macrophage-mediated inflammatory response triggered by metabolic disorders is a key link in protecting the kidneys.^[107]^

To survive the hyperglycemic, hypoxic, and inflammatory renal microenvironment, these immune cells undergo a profound metabolic reprogramming.^[108]^ This adaptive switch, however, is inherently maladaptive; it locks macrophages into a pro-inflammatory and pro-fibrotic phenotype that actively perpetuates tissue injury.^[109]^

#### Mitochondrial dysfunction

A breakdown in MQC, particularly the failure of mitophagy, is a central driver of macrophage pathogenicity in DKD.^[110]^ This inability to clear dysfunctional organelles results in the accumulation of ROS-generating mitochondria,^[111]^ locking the cell into a pro-inflammatory and pro-fibrotic state that directly correlates with declining glomerular filtration rates and progressive interstitial fibrosis.^[112]^ This causal link is substantiated by genetic evidence: macrophage-specific deletion of the mitophagy regulator Mitofusin 2 (MFN2) is sufficient to amplify ROS production and exacerbate the fibrotic response.^[113]^

#### Glycolysis

The pathological macrophage phenotype in DKD is driven by a pronounced metabolic switch to glycolysis, which fuels a pro-inflammatory (M1-like) state.^[114]^ While acute hyperglycemia can paradoxically steer monocytes toward an anti-inflammatory state by inhibiting Toll-like receptor signaling,^[115]^ the chronic and complex inflammatory milieu of DKD overwhelmingly favors their polarization into pathogenic M1 macrophages.^[116]^ This pro-inflammatory reprogramming is governed by key molecular hubs; for instance, the m6A demethylase FTO has been shown to promote M1 glycolysis *via* the Npas2/Hif-1*α* axis, cementing this pathway as a critical regulator of macrophage-driven renal injury.^[117]^

#### Hypoxia-inducible factor signaling

The HIF-1*α* signaling pathway is the central orchestrator of macrophage metabolic reprogramming in DKD.^[112]^ Activated by the characteristic renal hypoxia of the disease, HIF-1*α* drives the glycolytic switch required to sustain the pro-inflammatory M1 phenotype.^[118, 119]^ The activity of this pathway is itself subject to precise epigenetic control. Specifically, the m6A demethylase FTO acts *via* a Prrc2a-dependent mechanism to remove the m6A modification from Npas2 messenger RNA, which stabilizes Npas2 and potentiates Hif-1*α* signaling, thereby cementing the glycolytic and inflammatory state of M1 macrophages.^[112]^

#### Inflammation

Macrophage metabolic reprogramming orchestrates a pathogenic feedback loop that sustains chronic inflammation in DKD.^[116]^ The metabolically hyperactive, pro-inflammatory macrophage phenotype is a primary driver of renal fibrosis.^[120]^ In turn, the cytokines released by these cells dysregulate systemic and local lipid metabolism, creating a lipotoxic environment that further fuels macrophage polarization and activation, thus locking in the vicious cycle.^[121]^

Although acute hyperglycemia can paradoxically induce a transient anti-inflammatory monocyte response by suppressing Toll-like receptor signaling,^[122]^ this effect is decisively overridden by the complex, chronic inflammatory milieu of DKD, which perpetuates the pathogenic M1 state.

## Natural product-targeted regulation of metabolic reprogramming in DKD

**Figure 6:**
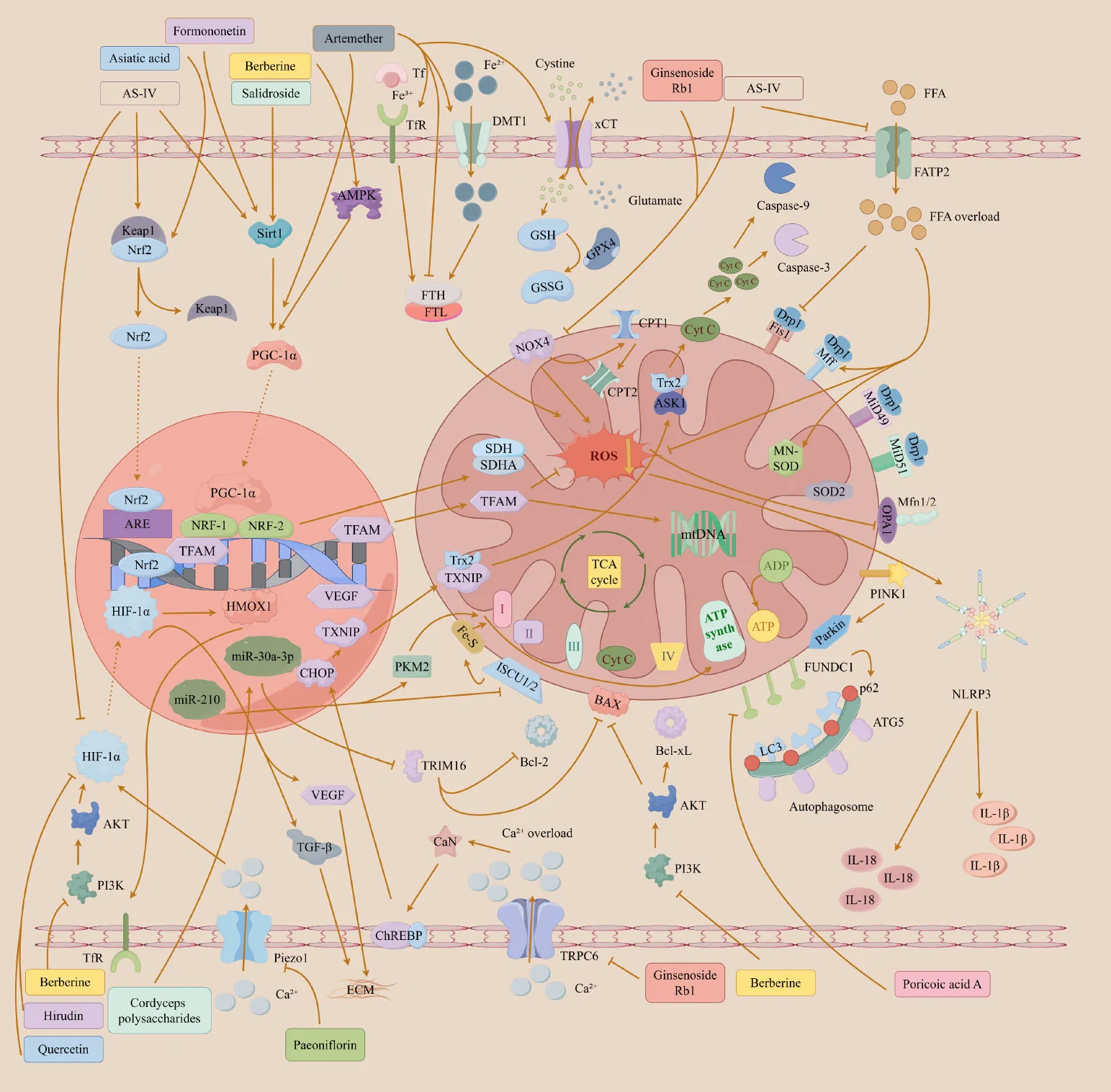
Mechanisms of natural products in regulating metabolic reprogramming for the treatment of diabetic kidney disease.

TCM is a complex system comprising a diverse range of chemical constituents, which reflects the rich biodiversity of Chinese medicinal plants.^[122, 123]^ Accumulating evidence indicates that various bioactive natural compounds can modulate glucose, lipid, and energy metabolism during the progression of DKD by regulating metabolic reprogramming.^[124]^ Such regulation may, in turn, alleviate pathological features including glomerulosclerosis and tubulointerstitial fibrosis.^[125]^ In this section, we systematically discuss the mechanisms of representative natural products—including glycosides, terpenoids, flavonoids, and alkaloids—thereby providing a theoretical foundation for the development of DKD-targeted therapeutic strategies based on metabolic reprogramming (Table 1, Figure 6).

**Table 1:** Application of natural active ingredients in renal metabolic reprogramming in diabetic kidney disease

Classification	Compound name	Model	Dosages	Time	*In vivo/* *In vitro*	Mechanism	Specific pathways	Molecular changes	References
	Astragaloside IV	HG-induced MPC5	3, 10, 20, 40, 80, 100 µmol/L	24 h	*In vitro*	Regulating mitochondrial dysfunction	Nrf2-ARE/TFAM signaling	↓ ROS ↑ HO-1, GCLC, GCLM, TFAM, Nrf1, PGC-1 α, TFAM, Nrf2, ETC complexes (CI, II, III, IV, V), ATP, GSH-Px, SOD2	[134]
		STZ-induced diabetes mice	6 mg/kg	10 wk	*In vivo*	Regulating mitochondrial dysfunction		↑ HO-1, GCLC, GCLM, TFAM, Nrf1, PGC-1α, TFAM, Nrf2, ETC complexes (CI, II, III, IV, V), ATP, GSH-Px, SOD2	
		STZ-induced diabetes rats	10, 20 mg/kg	8 wk	*In vivo*	Regulating mitochondrial dysfunction	inhibiting fatty acid transport protein-2	↓ Drp1, FATP2 ↑ MFN1, MN-SOD	[138]
		BSA-PA-treated NRK-52E cells	5, 10, 20 µmol/L	24/ 48 h	*In vitro*	Regulating mitochondrial dysfunction		↓ mtROS, Drp1, FATP2 ↑ MFN1, MN-SOD	
		db/db diabetic mice	20, 80 mg/kg	8 wk	*In vivo*	Regulating mitochondrial dysfunction	upregulating CPT1A- mediated HSD17B10 lysine succinylation	↑ complex I activity, NAD +/ NADH ratio	[217]
		HG-induced HK2 cells	40 µmol/L	72 h	*In vitro*	Regulating mitochondrial dysfunction		↓ ROS, NOX4, BAX ↑ PGC-1α, CPT1A, mitochondrial number, complex I activity, NAD + /NADH ratio, ATP	
		db/db diabetic mice	20 mg/kg	6 wk	*In vivo*	Regulating mitochondrial dysfunction	Nrf1/PGC1α /Nrf1 signaling	↑ Sirt1, PGC-1α, Nrf1, TFAM, mitochondrial morphology	[135]
		Phenylsulfate-induced podocytes	50, 100 µmol/L	24 h	*In vitro*	Regulating mitochondrial dysfunction		↓ mtROS ↑ CAT, HO-1, SOD2, Sirt1, PGC- 1α, Nrf1	
		STZ-induced diabetes rats	20, 40, 80 mg/kg	12 wk	*In vivo*	Regulating mitochondrial dysfunction	inhibit FTH1/ HMOX1/TFR1-medi- ated ferroptosis	↑ mitochondrial morphology	[135]
		HG-induced HK2 cells	25, 50, 100 µmol/L	24 h	*In vitro*	Regulating mitochondrial dysfunction		↓ mtROS ↑ MMP, ATP	
		db/db diabetic mice	1 g/kg	12 wk	*In vivo*	Regulating mitochondrial dysfunction	downregulated PINK1/Parkin-mediated mitophagy	↓ Drp1, Fis-1, MFF, PINK1, Parkin	[139]
		STZ-induced diabetes mice	25, 50, 100 mg/kg	12 wk	*In vivo*	Hypoxia	inhibiting HIF-1α/ HMOX1 pathway	↓ HIF-1 ↑ HMOX1	[142]
		HG-induced HK2 cells	100 µmol/L	24 h	*In vitro*	Hypoxia			
	Ginsenoside Rb1	STZ-induced diabetes mice	40 mg/kg	7 wk	*In vivo*	Regulating mitochondrial dysfunction	upregulated expression of aldose reductase (AR)	↓ Cyto c, Caspase 9, ROS	[151]
		HG-induced MPC5 cells	10 µmol/L	72 h	*In vitro*	Regulating mitochondrial dysfunction		↓ NOX4, Cyto c, Caspase 9, ROS	
	Ginsenoside Rg1	STZ-induced diabetes mice	10 mg/kg	8 wk	*In vitro*	Regulating mitochondrial dysfunction	inhibiting TRPC6-ChREBP-TXNIP signaling	↓ ROS	[152]
		PA and HG-induced HMCs	10 µmol/L	24 h	*In vitro*	Regulating mitochondrial dysfunction		↓ ASK1, TXNIP, cleaved caspase-3 ↑ Trx2, Cytochrome c	
	Salidroside	STZ-induced diabetes mice	50, 100 mg/kg	10 wk	*In vivo*	Regulating mitochondrial dysfunction	Sirt1/PGC-1α	↑ Nrf1/Ppia, PHB1, VDHc, SDHA, Cox IV, PGC-1α, Sirt1	[156]
	Paeoniflorin	db/db diabetic mice	50, 100 mg/kg	10 wk	*In vivo*	Hypoxia	targeting the Piezo1/Ca2+ /HIF-1α axis	↓HIF-1α	[161]

Terpenoids	Asiatic acid	STZ-induced diabetes rats	10, 30 mg/kg	10 wk	*In vivo*	Regulating mitochondrial dysfunction	regulating mitochondrial dynamics via the Nrf2 pathway	↓Drp1, Keap-1 ↑MFN1, MFN2, Nrf2, HO-1	[171]
		AGEs-induced HK2 cells	10, 20 µmol/L	24 h	*In vitro*	Regulating mitochondrial dysfunction		↓Drp1, Keap-1 ↑MFN1, MFN2, Nrf2, HO-1, mitochondrial morphology	
	Poricoic acid A	HG-induced MPC5 cells	3.125, 6.25, 12.5, 25, 50, 100, 200 µg/mL	24 h	*In vitro*	Regulating mitochondrial dysfunction	downregulating FUNDC1	↓p62, FUNDC1 ↑MMP, LC3, ATG5	[175]
		STZ-induced diabetes mice	10, 20 mg/kg	4 wk	*In vivo*	Regulating mitochondrial dysfunction		↓p62, FUNDC1 ↑LC3, ATG5	
	Artemether	db/db diabetic mice	0.67 g/kg	12 wk	*In vivo*	Regulating mitochondrial dysfunction	increasing mitochondrial pyruvate carrier content	↓H2O2 ↑PGC-1α, PDP1, MPC1,MPC2	[176]
		STZ-induced diabeetes mice	1.6 g/kg	8 wk	*In vivo*	Regulating mitochondrial dysfunction	regulate iron metabolism and resolution of mitochondrial damage	↓p-AMPK ↑mitochondrial cristae	[180]

Flavonoid	Quercetin	HG-induced MPC5 cells	10, 20, 40 µmol/L	72 h	*In vitro*	Glycolysis	HIF-1α/miR-210/ISCU/FeS Pathway	↓p-PKM2/PKM2	[187]
		HG-induced MPC5 cells	10, 20, 40 µmol/L	72 h	*In vitro*	Hypoxia	HIF-1α/miR-210/ISCU/FeS Pathway	↓HIF-1α, miR-210	[187]
	Formononetin	STZ-induced diabetes rats	20 mg/kg	8 wk	*In vivo*	Regulating mitochondrial dysfunction	Sirt1/PGC-1α	↓Drp1, Fis1 ↑mitochondrial morphology, MFN2, Sirt1, PGC-1α	[191]
		HG-induced HK2 cells	10, 20 µmol/L	48 h	*In vitro*	Regulating mitochondrial dysfunction		↓ROS, Drp1, Fis1 ↑MMP, MFN2, Sirt1, PGC-1α	

Alkaloids	Berberine	mouse podocytes	0.4 µmol/L	12 h	*In vitro*	Regulating mitochondrial dysfunction	increased the expression of AMPK, PPARs, and PGC-1α	GSH-Px activity and SOD2 ↑CTP1, CTP2, ACADL, ACADM, ACSL1, ACOX1, Slc27a2, MRC enzyme complexes I/IV/V	[200]
		db/db diabetic mice	200 mg/kg	8 wk	*In vivo*	Regulating mitochondrial dysfunction	promoting PGC-1α-regulated mitochondrial energy homeostasis	↑VDAC, CTP1, CTP2, ACADL, ACADM, ACSL1, ACOX1, Slc27a2, MRC enzyme complexes I/IV/V	
		mouse podocytes	0.4 µmol/L	12 h	*In vitro*	Regulating mitochondrial dysfunction	inhibit Drp1-mediated mitochondrial fission and dysfunction	↓mtROS, MFF, Fis1, Mid49, Mid51, Drp1 ↑MMP, ATP, PGC-1α, TFAM, Nrf1, Nrf2	[75]
		db/db diabetic mice	300 mg/kg	8 wk	*In vivo*	Regulating mitochondrial dysfunction		↓ Drp1, MFF, Fis1, Mid49, Mid51, mitochondrial fragmentation of podocytes	
		STZ-induced diabetes mice	100, 200 mg/kg	8 wk	*In vivo*	Regulating mitochondrial dysfunction	inhibits KLF4 promoter methylation and ferroptosis	↓ROS ↑mitochondrial morphology, GPX4	[118]
		NRK-52E and HK2 cells	0, 10, 30, 90 µg	72 h	*In vitro*	Hypoxia	activation of HIF-1α in the PI3K/Akt signal pathway	↓HIF-1α	[118]

Others	Cordyceps cicadae polysaccharides	db/db diabetic mice	75, 150, 300 mg/kg	12 wk	*In vivo*	Regulating mitochondrial dysfunction	miR-30a-3p/TRIM16	↑miR-30a-3p ↓TRIM16, 8-OHdG	[213]
		HG-induced MPC5 cells	100, 200, 400 µg/mL	24 h	*In vitro*	Regulating mitochondrial dysfunction		↓ TNF-α, IL-1β, IL-6, ROS	
	Hirudin	STZ-induced diabetes rats	1 ATU	16 wk	*In vivo*	Hypoxia	inhibiting HIF-1α/VEGF Signaling Pathway	↓HIF-1α	[216]
		HG-induced HK2 cells	160, 320 µg/mL	48 h	*In vitro*	Hypoxia		↓ HIF-1α/VEGF	

↑: increase; ↓: decrease; 8-OHdG: 8-hydroxy-2'-deoxyguanosine; ACADL: acyl-CoA dehydrogenase long chain; ACADM: acyl-CoA dehydrogenase medium chain; ACOX1: acyl-CoA oxidase 1; ACS: acyl-CoA synthetase; AMPK: AMP-activated protein kinase; ASK1: apoptosis signal-regulating kinase 1; ATG5: autophagy related 5; ATP: adenosine triphosphate; BAX: BCL2 associated X, apoptosis regulator; CAT: catalase; Cl: complex I; Cox IV: cytochrome c oxidase subunit 4; CPT1A: carnitine palmitoyltransferase 1A; CTP1: carnitine palmitoyltransferase 1; Cyto c: cytochrome c; Drp1: dynamin-related protein 1; ETC: electron transport chain; FATP2: fatty acid transport protein 2; Fis-1: mitochondrial fission 1 protein; FUNDC1: FUN14 domain containing 1; GCLC: glutamate-cysteine ligase catalytic subunit; GCLM: glutamate-cysteine ligase modifier subunit; GPX4: glutathione peroxidase 4; GSH-Px: glutathione peroxidase; H2O2: hydrogen peroxide; HG: high-glucose; HIF-1: hypoxia-inducible factor 1; HMOX1: heme oxygenase 1; HO-1: heme oxygenase 1; IL-1β: interleukin 1 beta; IL-6: interleukin 6; Keap-1: Kelch-like ECH-associated protein 1; LC3: microtubule associated protein 1 light chain 3; MFF: mitochondrial fission factor; MFN: mitofusin; Mid: mitochondrial dynamics protein; MMP: mitochondrial membrane potential; MPC: mitochondrial pyruvate carrier; MRC: mitochondrial respiratory chain; mtROS: mitochondrial reactive oxygen species; NOX4: NADPH oxidase 4; Nrf: nuclear respiratory factor; Parkin: parkin RBR E3 ubiquitin protein ligase; PDP1: pyruvate dehydrogenase phosphatase catalytic subunit 1; PGC-1α: peroxisome proliferator-activated receptor gamma coactivator 1-alpha; PHB1: prohibitin 1; PINK1: PTEN induced kinase 1; ROS: reactive oxygen species; SDHA: succinate dehydrogenase complex flavoprotein subunit A; Sirt1: sirtuin 1; Sirt2/3: sirtuin 2/3; Sirt6: sirtuin 6; SOD2: superoxide dismutase 2; STZ: streptozotocin; TFAM: mitochondrial transcription factor A; TNF-α: tumor necrosis factor alpha; TRIM16: tripartite motif containing 16; Trx2: thioredoxin 2; TXNIP: thioredoxin interacting protein; VDAC: voltage dependent anion channel; VEGF: vascular endothelial growth factor.

### Glycosides

Glycosides are naturally occurring secondary metabolites widely distributed in plants. Owing to the hydrolysis of glycosidic bonds, these compounds demonstrate considerable therapeutic potential and clinical applicability.^[126]^ Many bioactive molecules can conjugate with sugars or sugar derivatives to form glycosides, which are generally classified into oxygen glycosides (O-glycosides), sulfur glycosides (S-glycosides), nitrogen glycosides (N-glycosides), and carbon glycosides (C-glycosides).^[127]^ Its glycosyl portion usually greatly improves the water solubility of the parent glycoside, thus enhancing its bioavailability *in vivo*. The glycosidic bond itself can be specifically hydrolyzed by specific enzyme systems (*e.g*., *β*-glucosidase) in the gut or kidney, which corresponds to a targeted delivery mechanism: activated at the site of the lesion, the glycoside is released *in situ* with direct bioactivity, which in turn precisely exerts antioxidant, anti-inflammatory, or metabolic signaling pathway modulation.

#### Astragaloside IV

Astragaloside IV (AS-IV) is the most bioactive compound identified in *Astragalus*.^[128]^ Its oral absorption of the prototype drug is extremely poor; it requires hydrolysis by gut microbiota into its aglycone for absorption and is mainly excreted through the bile-feces route, resulting in extremely low oral bioavailability. It exerts anti-inflammatory, antioxidant, anti-fibrotic, anti-tumor, and neuroprotective effects by modulating multiple signaling pathways. For example, it combats inflammatory damage by regulating inflammatory factors (such as interleukin-1*β* (IL-1*β*), tumor necrosis factor-*α* (TNF-*α*), intercellular adhesion molecule, and chemokines), inflammatory mediators (such as nitric oxide (NO)), the nuclear factor kappa-light-chain enhancer of activated B cells (NF-*κ*B) signaling pathway, and apoptosis-related genes.^[129]^ One of the main mechanisms of AS-IV is its antioxidant activity. It delays or inhibits cellular oxidation by scavenging reactive oxygen species (ROS), or acts as an exogenous antioxidant to maintain or restore redox homeostasis.^[130]^ In terms of neuroprotection, it exerts significant protective effects against MPP^+^-induced cell death in SH-SY5Y cells by inhibiting the Bax/Bcl-2-related cell death pathway and ROS production.^[131]^ Furthermore, in renal proximal tubular cells, AS-IV attenuates oxidative stress by inhibiting glycation albumin-induced epithelial-mesenchymal transition, characterized by reduced expression of *α*-smooth muscle actin and increased expression of E-cadherin.^[132]^ It was shown that the protective effect of AS-IV against DKD may originate from the regulation of mitochondrial division.^[133]^ Shen *et al*.^[134]^ reported that podocytes exposed to high-glucose (HG) stimulation displayed mitochondrial morphological abnormalities, podocyte apoptosis, activation of ROS-induced oxidative stress responses, and downregulation of Nrf2, PGC-1*α*, and mitochondrial TFAM, which are key regulators of mitochondrial biogenesis and function. AS-IV treatment significantly reversed these pathological changes. Similarly, Liu *et al*.^[135]^ demonstrated that AS-IV activated Nrf1, exerting comparable protective effects in phenylsulfate-induced podocytes.

In HK-2 cells, AS-IV was shown to decrease lipid peroxides, Fe^2+^, and glutathione depletion, thereby repairing iron-related mitochondrial damage. It also reduced mitochondrial ROS, enhanced MMP, and increased ATP production, collectively counteracting mitochondrial dysfunction.^[136]^ Drp1, a core mediator of mitochondrial fission, is critical for maintaining mitochondrial morphology, function, and cellular homeostasis.^[137]^ Studies have shown that AS-IV markedly downregulated Drp1 expression in DKD rats and BSA-PA-treated NRK-52E cells, while reducing mitochondrial ROS in a dose-dependent manner.^[138]^ Consistently, Liu *et al*.^[139]^ found that the expression of Drp1, mitochondrial fission protein 1 (Fis1), and mitochondrial fission factor (MFF)—the major regulators of mitochondrial fission—was significantly elevated in db/db mice. AS-IV treatment effectively reversed their expression by downregulating PINK1/Parkin-mediated mitophagy. HIF-1*α*, a heterodimeric transcription factor, orchestrates cellular adaptation to hypoxia by regulating oxygen metabolism, glucose transport, and apoptosis.^[140, 141]^ Notably, AS-IV directly inhibited HIF-1*α* expression in STZ-induced diabetic mice and HG-induced HK-2 cells, while upregulating heme oxygenase 1 (HMOX1) to restore iron homeostasis. Through these actions, AS-IV improved the renal hypoxic microenvironment and conferred renal protection.^[142]^ Although most animal studies have shown that AS-IV improves mitochondrial function and attenuates pathological changes in DKD, there is a lack of large-scale randomized controlled clinical trials to validate its efficacy as of now. Systematic evaluations have been conducted pointing out that most of the available animal studies are small samples, short-term dosing, and scarce clinical translational data.^[143]^ Therefore, whether AS-IV is able to replicate the protective effects of animal models in the clinic still requires further validation.

#### Ginsenosides

Ginsenoside Rb1 is classified as a protopanaxadiol-type ginsenoside, whereas Ginsenoside Rg1 belongs to the protopanaxatriol-type subclass.^[144]^ Its oral absorption of the prototype ginsenosides is extremely low; their biological activity depends on metabolic conversion by gut microbiota into more readily absorbed aglycones (like compound K), leading to extremely low oral bioavailability for the prototypes. A recent review demonstrated that different subclasses of ginsenosides exert notable renoprotective effects, alleviating renal injury in models of nephritis, renal fibrosis, and diabetic kidney disease.^[145]^ Mitochondria have been identified as a primary target of Ginsenoside Rb1,^[146]^ through which it activates PINK1/Parkin-mediated mitophagy to clear damaged mitochondria, thereby reducing ROS production, inhibiting the downstream NLRP3/Caspase-1/GSDMD signaling pathway, and ultimately alleviating cell pyroptosis.^[147]^ Concurrently, *Ginsenoside* Rb1 significantly upregulates the mRNA abundance of key antioxidant genes, including SOD1, catalase (CAT), and GPx1. This upregulation in gene expression subsequently translates into an enhancement of protein function, manifested as a significant increase in the catalytic activity of endogenous antioxidant enzymes such as total superoxide dismutase, CAT, and glutathione peroxidase (GSH-Px), thereby establishing a more robust cellular antioxidant defense system.^[148]^ Furthermore, *Ginsenoside* Rb1 effectively stabilizes MMP by inhibiting the opening of the mitochondrial permeability transition pore and reducing ROS production.^[149]^ It was shown that Rb1 activates AMPK which in turn promotes the PGC-1*α*-Nrf1-TFAM axis driving mitochondrial biosynthesis and inhibits the NF-*κ*B-TGF-*β*1-fibrosis pathway and improve insulin resistance.^[150]^ Experimental studies have shown that Rb1 treatment reverses HG-induced increases in ROS, cytochrome c (Cyt c), caspase-9, and the mitochondrial regulatory protein NOX4.^[151]^ In parallel, Rg1 markedly attenuates mitochondrial injury and the release of pro-apoptotic proteins, while also reducing lipid deposition and ROS accumulation in T2DM models.^[152]^ These findings indicate that Ginsenosides ameliorate renal metabolic reprogramming by alleviating mitochondrial dysfunction.

#### Other glycosides

Salidroside is the principal bioactive component isolated from the roots of *Rhodiola rosea*.^[153]^ As a small-molecule phenylethanoid glycoside, it is rapidly absorbed and cleared, primarily excreted *via* the kidneys, representing a glycoside with relatively high oral bioavailability. Owing to the hydroxyl group on its aglycone, it is categorized as an alcohol glycoside within the oxygen glycoside class, as it is linked to glucose *via* an oxygen atom.^[154]^ Previous studies have shown that salidroside attenuates oxidative stress and inflammation in STZ-induced diabetic rats by activating the Akt/GSK-3*β* signaling pathway, thereby conferring renoprotective effects.^[155]^ Furthermore, research has demonstrated that salidroside restores Sirt1 expression, leading to the activation of PGC-1*α*, regulation of mitochondrial function, and subsequent alleviation of renal injury in STZ-induced diabetic mice.^[156]^ However, in GK type 2 diabetic rats, Salidroside did not reduce blood pressure, blood glucose or insulin resistance, and had only a localized effect on vasodilation.^[157]^ This suggests that *Salidroside* may participate in DKD metabolic reprogramming by interfering with mitochondrial function, but its metabolic modulatory effects may be affected by model differences and its clinical efficacy needs to be further evaluated.

Total glycosides of *Paeonia* (TGP) are extracted from the dried roots of *Paeonia lactiflora* Pallas.^[158]^ Due to its strong hydrophilicity, its oral absorption is poor and highly dependent on gut microbiota to be metabolized into its aglycone before absorption, resulting in very low oral bioavailability. Paeoniflorin is the major bioactive constituent of TGP and has been reported to ameliorate macrophage infiltration and activation by inhibiting the TLR4 signaling pathway in DKD.^[159]^ Paeoniflorin inhibits renal inflammation by promoting macrophage polarization from M1 to M2 and inducing mitochondrial autophagy by modulating KLF4.^[160]^ Moreover, paeoniflorin directly targets the Piezo1/Ca^2+^/HIF-1*α* axis, thereby suppressing the upregulation of HIF-1*α*-mediated proteins. Through this mechanism, it participates in the metabolic reprogramming of DKD by ameliorating renal hypoxia.^[161]^

### Terpenoids

Terpenoids are a diverse class of natural organic compounds that are widely distributed in plants. Their structures are derived from isoprene units (C_5_H_8_) as the fundamental building blocks, polymerized predominantly through head-to-tail linkages.^[162]^ Based on the number of isoprene units, terpenoids are categorized into monoterpenes, sesquiterpenes, diterpenes, triterpenes, and other subclasses, reflecting substantial structural diversity and a wide range of biological activities.^[163]^ In recent years, terpenoids have been extensively investigated for their potential roles in anticancer through the two core mechanisms of inducing apoptosis and modulating autophagy, and by involving multiple key signaling pathways such as PI3K/AKT/mTOR, MAPK, and ROS.^[164]^ The anti-inflammatory effects of terpenoids are primarily achieved by inhibiting key pro-inflammatory enzymes, such as inducible nitric oxide synthase, cyclooxygenase, and lipoxygenase, thereby reducing the production of NO, prostaglandins, and pro-inflammatory cytokines, including TNF-*α* and various interleukins. The key pathways for their anti-inflammatory action mainly cover the inhibition of NF-*κ*B activation, nuclear translocation, and regulated transcriptional activity, as well as the modulation of the MAPK (including ERK, JNK, and p38) signaling pathway.^[165]^ In the treatment of diabetes, terpenoids reduce intestinal glucose absorption by inhibiting the SGLT-1 cotransporter and *α*-glucosidase/*α*-amylase, and they promote glucose uptake in tissues by regulating GLUTs transporters. Intracellularly, terpenoids can activate key signaling pathways such as PI3K/Akt and AMPK while inhibiting the activity of PTP1B phosphatase, thereby enhancing insulin signal transduction and sensitivity. Additionally, they can alleviate complications caused by hyperglycemia through the inhibition of aldose reductase and mitigate diabetes-related pathological damage *via* antioxidant and anti-inflammatory effects (such as inhibiting the NF-*κ*B factor).^[166]^

#### Asiatic acid

Asiatic acid (AA) is a triterpenoid compound extracted from *Centella asiatica*.^[167]^ As a pentacyclic triterpene, its extremely poor water solubility is the main reason for its limited oral absorption and very low bioavailability. It has been reported to exert protective effects on renal podocytes in diabetic rats.^[168]^ Mitochondria, as double-membrane organelles, are central to cellular energy metabolism and play a critical role in regulating cell survival and death.^[169, 170]^ Evidence from STZ-induced diabetic rats and AGEs-induced HK-2 cells indicates that AA regulates the Nrf2 pathway, thereby suppressing Drp1 expression, upregulating MFN1/2, and ultimately ameliorating renal tubular and mitochondrial injury by restoring mitochondrial fusion.^[171]^

#### Poricoic acid A

Poricoic acid A is a major triterpenoid component of *Poria cocos* and has been shown to exert significant antifibrotic effects in the kidney.^[172, 173]^ As a tetracyclic triterpene, its main barrier to absorption is also poor water solubility, and therefore its oral bioavailability is expected to be very low. FUN14 domain-containing protein 1 (FUNDC1), located on the outer mitochondrial membrane, functions as a receptor for hypoxia-induced, receptor-mediated mitophagy by interacting with LC3, thereby playing a critical role in maintaining mitochondrial homeostasis.^[174]^ Wu *et al*.^[175]^ reported that poricoic acid A isolated from *Poria cocos* markedly increased LC3 and ATG5 expression in HG-induced MPC5 cells and kidney tissue from DKD mice, while simultaneously reducing p62 and FUNDC1 levels. These changes collectively activated mitochondrial autophagy and consequently alleviated podocyte injury.

#### Artemether

Artemether, as a prodrug, although it can be absorbed orally, it undergoes an extremely rapid and extensive first-pass effect, being efficiently converted into the active metabolite dihydroartemisinin, which results in low bioavailability of the parent drug. A sesquiterpene derivative of artemisinin, has been shown to upregulate MPC1/2 expression by targeting PGC-1*α*, thereby reducing mitochondrial H_2_O_2_ release in db/db diabetic mice.^[176]^ Iron, an essential element for human physiology, participates in multiple processes, with iron-containing proteins being crucial for oxygen transport, storage, and utilization. Since mitochondria are the primary site of oxidative phosphorylation, they are intimately linked to iron metabolism.^[177, 178]^ Evidence from a Mendelian randomization study demonstrated a significant positive correlation between systemic iron overload and type 2 diabetes.^[179]^ Furthermore, artemether has been reported to correct iron metabolism abnormalities in STZ-induced diabetic mice and improve mitochondrial ultrastructure.^[180]^ Whereas iron metabolism is a process closely linked to mitochondrial oxidative phosphorylation and metabolic homeostasis, this is likely a reflection of its broader, aimed at restoring mitochondrial integrity and normalizing metabolic function. Collectively, these findings suggest that terpenoid herbal compounds, such as artemether, may exert therapeutic potential in DKD by simultaneously targeting mitochondria and their associated regulatory networks.

### Flavonoids

Flavonoids are low-molecular-weight metabolites of natural plant polyphenols, predominantly found in vascular plants. They exhibit diverse pharmacological and biological activities.^[181]^ As a dietary component, flavonoids contribute to the maintenance of human health.^[182]^ Flavonoids can act synergistically through multiple targets—elevating insulin signaling, activating AMPK-mTOR, upregulating Nrf2/HO-1 antioxidant, inhibiting NF-*κ*B inflammation, and modulating SIRT1-PGC-1*α* mitochondrial function—systematically restores glucose and lipid metabolism, thereby realizing the metabolic reprogramming regulation of diabetic nephropathy.^[183]^

#### Quercetin

Quercetin is one of the most potent antioxidants among flavonoids and is recognized for its safety and minimal side effects.^[184]^ Due to its poor solubility and extremely extensive and rapid phase II conjugation metabolism in the intestine and liver, the oral bioavailability of its prototype form is extremely low. It has been approved for use as an antioxidant, an anti-allergic agent, and as a supplement for the COVID-19 Delta variant.^[185, 186]^ Xu *et al*.^[187]^ reported that quercetin inhibits HIF-1*α* and miR-210 expression while downregulating p-PKM2/PKM2 in a dose-dependent manner, thereby alleviating aerobic glycolysis in HG-induced mice glomerular mesangial cells. Nevertheless, its low bioavailability remains a major obstacle to its development as a therapeutic agent.^[188]^

#### Formononetin

Formononetin is primarily found in leguminous plants and certain traditional Chinese medicines.^[189]^ As a relatively well-absorbed isoflavone, its oral bioavailability is moderate but is still limited by gut microbiota and phase II conjugation metabolism. Previous studies have reported that formononetin can improve mitochondrial dysfunction-related targets in cardiomyocytes and alleviate cardiac fibrosis induced by *β*-adrenergic activation.^[190]^ As research on this compound and mitochondrial biology continues, studies in STZ-induced diabetic rats and HG-induced HK-2 cell models have shown that formononetin restores the expression of Drp1, Fis1, and MFN2, as well as apoptosis-related proteins, including Bax, Bcl-2, and cleaved caspase-3, and MMP loss, thereby exerting a renal protective effect.^[191]^ This demonstrates that Formononetin can modulate renal metabolic reprogramming through the mitigation of mitochondrial dysfunction, thereby exerting a renal protective effect.

### Alkaloids

Alkaloids are among the largest classes of plant secondary metabolites, typically derived from amino acids and containing one or more nitrogen atoms in their structure, and they are particularly abundant in angiosperms.^[192]^ In TCM, alkaloids represent important pharmacologically active substances, and systematic research on taxonomic groups rich in bioactive alkaloids can facilitate the development and identification of new drugs.^[193]^ A review summarized the molecular forms of alkaloids extracted and isolated from various species, and experimental studies in animal models, as well as clinical trials in humans, have demonstrated their efficacy in ameliorating elevated blood glucose levels.^[194]^

#### Berberine

Berberine is an isoquinoline alkaloid isolated from traditional Chinese herbs such as *Coptis chinensis*. Due to its weak membrane permeability and being a strong substrate for the P-glycoprotein efflux pump, its oral absorption and bioavailability are extremely low, although it accumulates significantly in tissues. It is commonly used as an antibacterial agent for treating diarrhea and other gastrointestinal infections.^[195, 196]^ Recent reports indicate that berberine can improve diabetes in metabolic disorder states by regulating glucose and lipid metabolism, demonstrating its activity as an antidiabetic agent.^[197]^ The mechanisms by which berberine intervenes in diabetic kidney disease have become a current research focus.^[198, 199]^ In 2019, Qin *et al*.^[75]^ first demonstrated that berberine may exert protective effects on glomeruli and podocytes by upregulating Drp1-mediated mitochondrial dynamics, downregulating MFF, Fis1, Mid49, and Mid51, and restoring MMP and ATP levels. Subsequently, they used metabolomics technology to show that mitochondrial dynamics are altered in DKD patients, accompanied by widespread mitochondrial dysfunction. *In vivo* and *in vitro* experiments confirmed that berberine inhibits excessive mitochondrial ROS production and functional impairment in DKD mouse models and podocytes, primarily through activation of the PGC-1*α* signaling pathway, thereby promoting mitochondrial energy homeostasis in podocytes.^[200]^ Kruppel-like factor 4 (KLF4) is an evolutionarily conserved zinc finger transcription factor that regulates various cellular processes, including growth, proliferation, and differentiation.^[201]^ Since its discovery in 1996, KLF4 has attracted significant attention, and in 2006, it was identified as one of the four factors required for inducing pluripotent stem cells.^[202]^ Increasing evidence suggests that members of the KLF family play crucial roles in mitochondrial maintenance during biogenesis, dynamics, and mitophagy across multiple organ systems.^[203, 204, 205]^ Cai *et al*.^[206]^ reported that berberine can upregulate KLF4 expression by blocking STZ-induced promoter methylation in mice, thereby restoring mitochondrial function. Recent studies using endothelium-specific KLF4 knockout mice to model thrombotic microangiopathy, along with biopsy specimens from patients showing reduced KLF4 and CD55 levels, demonstrated that KLF4 regulates complement deposition in glomeruli,^[207]^ highlighting its therapeutic relevance. Additionally, berberine protects renal tubular epithelial cells from HG-induced apoptosis by activating HIF-1*α* *via* the PI3K/Akt signaling pathway, strongly suggesting that berberine may be a potential therapeutic agent for DKD.^[208]^

### Others

Based on the chemical structure of the natural products, natural products that have been studied in relatively small numbers but have unique mechanisms of action (cordyceps polysaccharides, hirudin) are categorized in this paper. Preliminary and convincing evidence for the mechanism of action of these products has been obtained, but the relevant studies are still relatively limited compared to those compounds that have been more extensively studied in the previous chapters.

#### Cordyceps polysaccharides

Polysaccharides are high-molecular-weight polymers composed of ten or more monosaccharides, such as glucose, galactose, and mannose, linked by glycosidic bonds.^[209]^ As macromolecules, they are barely absorbed into the bloodstream after oral administration, leading to a prototype bioavailability of zero; they mainly exert prebiotic and immunomodulatory effects within the gut. They exhibit biological activities, including water solubility and low toxicity, and are widely distributed.^[210]^ Common examples include *Astragalus* polysaccharides, *Lycium* polysaccharides, *Ganoderma lucidum* polysaccharides, *Ginseng* polysaccharides, and *Poria cocos* polysaccharides. Cordyceps polysaccharides are the primary active components of Cordyceps and have been shown to inhibit inflammatory responses and regulate intestinal microbiota dysbiosis by blocking the TLR4/NF-*κ*B and TGF-*β*1/Smad signaling pathways, thereby exerting protective effects against renal tubulointerstitial fibrosis in DKD rats.^[211]^ TRIM16, a member of the Tripartite Motif family of proteins, regulates the formation and degradation of protein aggregates by modulating the Nrf2 and autophagy cellular detoxification systems, serving as a key hub linking protein quality control and mitochondrial homeostasis.^[212]^ Zheng *et al*.^[213]^ reported that *Cordyceps cicadae* polysaccharides can directly target TRIM16, the downstream gene of miR-30a-3p, and inhibit its expression, thereby exerting a protective effect on HG-induced MPC5 cells.

##### Hirudin

Hirudin is an acidic polypeptide secreted by the salivary glands of leeches and is the most potent naturally occurring specific inhibitor of thrombin discovered to date.^[31]^ Previous studies have reported that hirudin can ameliorate Gsdmd-mediated pyroptosis by inhibiting interferon regulatory factor 2 (Irf2), thereby alleviating kidney injury,^[214]^ suggesting that hirudin may serve as a potential therapeutic strategy for DKD. Transient hypoxia and activation of the HIF-1*α* pathway promote tissue repair; however, in DKD and renal tubular hypoxia, HIF-1*α* rapidly accumulates in cells, leading to increased expression of vascular endothelial growth factor (VEGF) and its receptor VEGF-R2.^[215]^ Hirudin can reduce the levels of ECM markers by inhibiting the HIF-1*α*/VEGF signaling pathway in DKD renal tubular epithelial cells.^[216, 217, 218]^ These results demonstrate that Hirudin ameliorates renal metabolic reprogramming in DKD by alleviating renal hypoxia.

## Conclusions and Perspectives

The pathogenesis of DKD is complex and involves metabolic reprogramming of multiple renal cell types, including renal tubular epithelial cells, podocytes, mesangial cells, endothelial cells, and macrophages. This reprogramming is primarily characterized by mitochondrial dysfunction, enhanced glycolysis, lipid accumulation, and aberrant activation of the HIF signaling pathway. Collectively, these metabolic disturbances promote renal inflammation, fibrosis, and functional decline, ultimately resulting in irreversible renal damage.

This review systematically elucidates the mechanisms by which various natural products, including glycosides, terpenoids, flavonoids, alkaloids, polysaccharides, and peptides, exert renal protective effects in DKD models by modulating key nodes of metabolic reprogramming, such as Drp1-mediated mitochondrial fission, PGC-1*α*-associated mitochondrial biogenesis, Sirt3 deacetylation activity, and the HIF-1*α* signaling pathway. These natural products offer multi-targeted actions with low toxicity and can simultaneously enhance mitochondrial function, inhibit glycolytic reprogramming, and alleviate oxidative stress and inflammatory responses, thereby providing novel therapeutic strategies for DKD.

Despite demonstrating substantial potential in preclinical studies, natural products still face significant challenges in clinical translation for DKD. First, most studies are limited to cellular and animal models, with a lack of large-scale clinical trials to confirm their safety and efficacy. Second, pharmacokinetic properties, bioavailability, and tissue-specific delivery of natural products remain inadequately addressed. Furthermore, DKD patients exhibit considerable heterogeneity, highlighting the need to further investigate the relationship between metabolic phenotypes and the therapeutic efficacy of natural products to enable personalized treatment. In addition, the preclinical studies included in this review relied mainly on animal models such as STZ induction and db/db, where the STZ model lacks the insulin resistance background of type 2 diabetes and the db/db model struggles to develop the progressive renal failure and significant renal fibrosis typical of advanced human DKD, and there are inherent limitations in reproducing the full picture of human DKD in these models, and these differences may have resulted in the therapeutic effects of natural products are overestimated in preclinical models and are one of the key factors contributing to the low success rate of translating basic research discoveries to the clinic.

Future research should prioritize elucidating the molecular mechanisms through which natural products regulate metabolic reprogramming. This can be achieved by employing advanced techniques, including multi-omics approaches and single-cell sequencing, to systematically analyze their target molecules and pathway networks. Simultaneously, efforts should focus on optimizing the structural design of natural products and developing delivery systems, using strategies such as nanotechnology and liposomes to improve bioavailability and renal targeting. Moreover, future studies need to be validated in models that better mimic the complex pathological processes of human DKD, rigorously designed clinical studies are required to validate their safety and efficacy, and to explore potential synergistic effects with existing standard therapies, such as SGLT2 inhibitors and ACEI/ARB, thereby establishing a novel, multi-modal, and personalized therapeutic strategy for DKD.

In summary, this study systematically reviewed the preclinical evidence of natural products against metabolic reprogramming for DKD intervention, providing a solid theoretical basis and direction for future targeted clinical trials prioritizing the most promising candidate molecules, designing rational drug combination regimens, and searching for precise biomarkers. Through interdisciplinary collaboration and translational research, it is anticipated that future breakthroughs will bridge basic research and clinical application, ultimately offering DKD patients safer and more effective treatments.
